# Early intrauterine pregnancy with an intrauterine device in place and terminated with spontaneous abortion: A case report

**DOI:** 10.1097/MD.0000000000037843

**Published:** 2024-04-19

**Authors:** Yu-Qun Chu, Chin-Tzu Tien, Dah-Ching Ding

**Affiliations:** a Department of Obstetrics and Gynecology, Hualien Tzu Chi Hospital, Buddhist Tzu Chi Medical Foundation, Tzu Chi University, Hualien, Taiwan; b Institute of Medical Sciences, Tzu Chi University, Hualien, Taiwan.

**Keywords:** abortion, case report, contraception, ectopic pregnancy, intrauterine device, intrauterine pregnancy

## Abstract

**Rationale:**

The overall pregnancy rate in individuals with an intrauterine device (IUD) for contraception is <1%. If pregnancy occurs while an IUD is in place, there is a higher risk of an ectopic pregnancy. We report the case of a woman with an IUD who was 7 weeks pregnant and experienced a spontaneous abortion 1 week later.

**Patient concern:**

A 32-year-old woman presented to our outpatient department with intermittent vaginal staining for several days.

**Diagnoses:**

She was 7 weeks pregnant and had an IUD in place for over 4 years. A vaginal examination revealed no vaginal bleeding and no blood clots; however, a parous cervix was observed. The IUD string was not visible. Transvaginal ultrasonography revealed a gestational sac in the uterine cavity, with a fetal pole and a crown-rump length of 11.4 mm. The fetal heart rate was 159 beats/min. The IUD was located in the retroplacental region. The bilateral adnexa appeared normal (right ovary, 2.9 cm; left ovary, 2.5 cm). The patient was diagnosed with an intrauterine pregnancy with an IUD in place and threatened abortion.

**Interventions:**

Attempts to remove the IUD were abandoned due to its location, and conservative treatment was initiated with Utrogestan (100 mg) administered 3 times a day for 1 week. Bed rest was advised.

**Outcomes:**

Unfortunately, she experienced a complete abortion 1 week later.

**Lessons:**

The novelty of this case report lies in the rare occurrence of an intrauterine pregnancy with a long-term IUD in place, the challenges posed by the IUD’s specific location, and the complex management of threatened abortion in this context. Our case highlights the diagnostic management approach for intrauterine pregnancy with an IUD in place. Furthermore, it explores the impact of IUD location on pregnancy prognosis.

## 1. Introduction

Intrauterine devices (IUDs) are widely used for contraception, with the 2 primary types being the T-shaped copper IUD and the similarly shaped levonorgestrel-releasing IUD. The overall pregnancy rate associated with IUD use for contraception is <1%. In such cases, pregnancies can be ectopic or intrauterine. However, IUDs are more effective at preventing intrauterine implantation. Thus, if pregnancy occurs while an IUD is in place, there is a higher likelihood of it being ectopic.^[1]^

The coexistence of an intrauterine pregnancy with an IUD is a relatively uncommon occurrence and is associated with an increased likelihood of adverse outcomes. Potential complications include late miscarriage, preterm delivery, vaginal bleeding, clinical chorioamnionitis, and placental abruption.^[2]^ Notably, suppose a woman chooses to continue with the pregnancy. In that case, it has been found that removing an IUD with visible strings or an IUD that is located within the cervix significantly improves pregnancy outcomes compared to leaving the IUD in place. Thus, if a woman decides to maintain her pregnancy, she should be adequately counseled about the increased risk of potential complications.^[3,4]^

Here, we report the case of a woman with an IUD who was 7 weeks pregnant and experienced spontaneous abortion 1 week later. Written informed consent was obtained from the patient.

## 2. Case presentation

### 2.1. Chief complaints

Intermittent vaginal staining for several days.

### 2.2. History of present illness

A 32-year-old woman presented to our outpatient department with a history of intermittent vaginal staining for several days. She was 7 weeks pregnant and concurrently had an IUD that had been in place for over 4 years.

### 2.3. History of past illness

She experienced intermittent lumbago 1 year prior, and lumbar spondylosis was suspected.

### 2.4. Personal and family history

She had 1 child (10 years old) that was delivered vaginally.

### 2.5. Physical examination

Vaginal examination revealed no evidence of vaginal bleeding or blood clots. Her cervix appeared parous, and the IUD string was not visible.

### 2.6. Laboratory examinations

Laboratory examinations were not performed.

### 2.7. Imaging examinations

**Figure 1.**
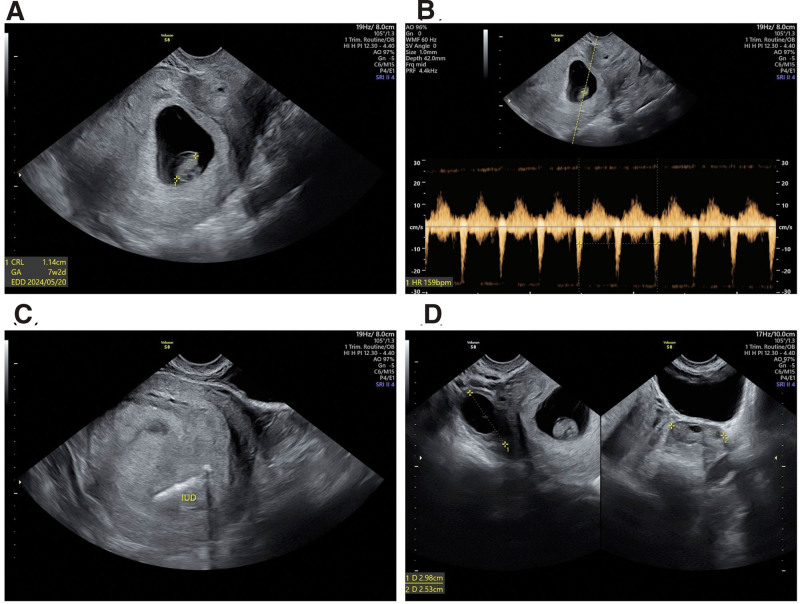
Transvaginal ultrasound of the pregnancy with an IUD in place. (A) Crown-rump length of the fetus. (B) Fetal heartbeat of 159 bpm. (C) The IUD is located in the placental region. (D) Bilateral adnexa are in normal appearance.

Transvaginal ultrasonography revealed a gestational sac in the uterine cavity with a fetal pole and a crown-rump length of 11.4 mm (Fig. 1A). The fetal heart rate was 159 beats/min (Fig. 1B). The IUD was located in the retroplacental region (Fig. 1C). The bilateral adnexa appeared normal (right ovary, 2.9 cm; left ovary, 2.5 cm) (Fig. 1D).

## 3. Final diagnosis

Intrauterine pregnancy at 7 weeks with an IUD in place, accompanied by a threatened abortion.

## 4. Treatment

As the IUD was located in the retroplacental region above the cervix, attempts to remove it were abandoned. The patient was administered Utrogestan (100 mg) 3 times a day for 1 week, and bed rest was advised.

## 5. Outcome and follow-up

She visited the emergency department because of massive vaginal bleeding 5 days later. Pelvic ultrasonography revealed a fetal pole with a heartbeat. Vital signs indicated a blood temperature of 36.2 °C, blood pressure of 104/69 mm Hg, pulse rate of 85 bpm, and respiratory rate of 20/min. No cervical dilation was observed during the examination. Progesterone 25 mg and tranexamic acid 1000 mg were administered by intramuscular and intravenous injection, respectively. After the vaginal bleeding decreased, she returned home and was prescribed tranexamic acid (250 mg) 3 times daily for 3 days.

Unfortunately, she experienced massive vaginal bleeding 2 days later. Upon examination, the uterine cervix had dilated with gestational tissue. The tissue was removed and sent for pathological examination, and the IUD was simultaneously removed. Histopathological examination revealed a decidual endometrium with focal chorionic villi. She was prescribed acetaminophen 500 mg 4 times a day, ferric hydroxide polymaltose 100 mg tablets once daily, and ergometrine maleate 0.2 mg twice daily for 1 week.

One week later, she was followed up without reporting any specific complaints.

## 6. Discussion

The novelty of this case report lies in the rare occurrence of an intrauterine pregnancy with a long-term IUD in place, the challenges posed by the IUD’s specific location, and the complex management of threatened abortion in this context. The case contributes to the medical literature by providing insights into the diagnosis, treatment, and outcomes of such unique and challenging scenarios.

In recent years, IUDs have gained increasing popularity as a contraceptive method due to their safety, efficacy, ease of use, and cost-effectiveness.^[5]^ The failure rate associated with IUD placement is comparable to that of tubal sterilization, with the highest risk of pregnancy occurring within the first year, ranging from 0.2 to 0.8%.^[6]^ Previous IUD expulsion is significantly linked to the risk of IUD failure. Age is another factor associated with IUD failure, with a significantly reduced risk in women aged > 35 years. Other factors, such as gynecological history and medication use, are not associated with IUD failure.^[7]^ In our case, the patient was 32 years old, and the IUD had been in place for over 4 years, suggesting a higher likelihood of IUD failure than in those >35 years old.

While IUDs are highly effective in preventing pregnancy, they are not foolproof, and conception can still occur. The symptoms and signs of pregnancy with an IUD are generally the same as those without an IUD, including missed periods, nausea and vomiting, headaches, sore and enlarged breasts, tiredness or fatigue, and cramping.^[8]^ When diagnosing pregnancy with an IUD, healthcare providers must exercise caution to prevent unintentional cognitive biases from influencing their diagnostic decisions. Practitioners should be aware of confirmation bias, which involves interpreting information to align with preexisting beliefs; the affect heuristic, which can influence decisions based on emotional reactions; and anchoring bias, which assigns undue importance to initial impressions, even if they may not be entirely valid.^[9]^ In cases where pregnancy with an IUD is suspected, diagnostic tools such as urine pregnancy tests, beta-human chorionic gonadotrophin serum testing, and diagnostic ultrasound can be utilized to assess pregnancy status.^[1]^ It is essential to first rule out ectopic pregnancy, especially in cases with IUDs.^[10]^ In our patient, ultrasonography confirmed an intrauterine pregnancy.

Subsequent steps following a positive pregnancy test indicating intrauterine pregnancy depend on various factors, including the woman’s decision regarding pregnancy continuation or termination, gestational age, IUD position, and visualization of the IUD string.^[10]^ For those seeking pregnancy termination, IUD removal can be performed during surgical abortion or before a medication-induced abortion. In cases where the patient wishes to continue their pregnancy, a pelvic examination should be conducted. If the IUD string is visible, gentle traction can be used for its removal. However, if the IUD string is not visible, its position should be confirmed using pelvic ultrasonography. In our case, the IUD was located within the cervix and was subsequently removed. However, if the IUD is positioned above the cervix, attempts at removal should be avoided. Women who desire to continue their pregnancy should be informed about the risks of obstetric complications associated with continuing their pregnancy while using an intrauterine IUD. Whether the decision is to continue or terminate the pregnancy in cases where the IUD remains unlocatable, confirmation of the IUD position should be postponed until pregnancy resolution. Abdominal and pelvic X-ray imaging can be used to locate the IUD.^[4,10]^ In our case, since the patient decided not to undergo an induced abortion, and the IUD located in the placental region could not be removed, she was informed about the increased risk of obstetric complications. These complications include spontaneous abortion, preterm delivery, septic abortion, and chorioamnionitis. Even after IUD removal, the risks remain higher than those in pregnancies without an IUD.^[11]^ Ultimately, consistent with the literature, the patient’s pregnancy ended in spontaneous abortion.

Ultrasonography is the preferred method for precisely locating IUDs owing to its cost-effectiveness and avoidance of ionizing radiation. Ultrasonography also provides a detailed description of the pelvic anatomy. On transvaginal ultrasonography, IUD stems appear as linear echogenic structures, with fully echogenic copper IUD arms and levonorgestrel-releasing IUD arms and echogenic proximal and distal ends with specific acoustic features. When IUDs are not visible on pelvic ultrasonography, X-rays can be used because all IUDs are radiopaque.^[12]^ The ultrasonography revealed that the IUD stem had a linear echogenic structure.

In other case reports of singleton intrauterine pregnancies with an IUD in situ in an otherwise normal uterus, the position of the IUD varied among patients. One study reported that the IUD was situated below the gestational sac, whereas in another case, it was positioned at the anterior wall of the uterus. Moreover, some pregnancies proceed without the IUD being detected during pregnancy but were discovered within fetal membranes during delivery.^[13–15]^ Notably, in all these case reports, the pregnancies progressed normally, and the patients successfully delivered healthy infants without congenital abnormalities. In contrast, our case involved a patient who was pregnant with an IUD located in the retroplacental region and experienced a miscarriage in the 8th gestational week.

A retrospective cohort study suggested that removing an IUD positioned within the uterine cavity did not significantly affect pregnancy outcomes. However, it is essential to note that retaining the IUD in a low-lying position is associated with an increased risk of miscarriage and adverse pregnancy outcomes compared with IUD removal. It is worth noting that there is a paucity of case studies on both groups of women with retained IUDs in the uterine cavity and low-lying positions; therefore, larger-scale case studies that comprehensively assess the impact of IUD location on pregnancy outcomes are needed in the future.^[16]^

## 7. Conclusion

This case report describes a rare scenario of a 32-year-old woman with a 7-week intrauterine pregnancy coexisting with a 4-year-old IUD. The IUD, located in the retroplacental region, posed challenges in management. Despite attempts to remove the IUD, it was abandoned due to its position above the cervix. The patient experienced a threatened abortion, leading to intermittent vaginal bleeding. Treatment involved Utrogestan, bed rest, and subsequent interventions with progesterone and tranexamic acid. Despite initial improvement, the patient later presented with massive vaginal bleeding, leading to the dilation of the cervix. Histopathological examination revealed a decidual endometrium with focal chorionic villi. The IUD was successfully removed, and the patient was managed with medications. Follow-up 1 week later showed no specific complaints. The case underscores the complexities of managing concurrent intrauterine pregnancy and IUD, offering insights into diagnostic and therapeutic challenges.

## Author contributions

**Conceptualization:** Dah-Ching Ding.

**Data curation:** Yu-Qun Chu, Chin-Tzu Tien, Dah-Ching Ding.

**Formal analysis:** Dah-Ching Ding.

**Writing – original draft:** Yu-Qun Chu, Chin-Tzu Tien, Dah-Ching Ding.

**Writing – review & editing:** Dah-Ching Ding.
